# Surface hydrophobicity and acidity effect on alumina catalyst in catalytic methanol dehydration reaction

**DOI:** 10.1002/jctb.5371

**Published:** 2017-09-08

**Authors:** Ahmed I Osman, Jehad K Abu‐Dahrieh, David W Rooney, Jillian Thompson, Samih A Halawy, Mohamed A Mohamed

**Affiliations:** ^1^ School of Chemistry and Chemical Engineering Queen's University Belfast Belfast Northern Ireland UK; ^2^ Chemistry Department, Faculty of Science – Qena South Valley University Qena Egypt

**Keywords:** DME, methanol dehydration, mesoporous alumina, boehmite, Cu/Al_2_O_3_

## Abstract

**BACKGROUND:**

Methanol to dimethyl ether (MTD) is considered one of the main routes for the production of clean bio‐fuel. The effect of copper loading on the catalytic performance of different phases of alumina that formed by calcination at two different temperatures was examined for the dehydration of methanol to dimethyl ether (DME).

**RESULTS:**

A range of Cu loadings of (1, 2, 4, 6, 10 and 15% Cu wt/wt) on Al_2_O_3_ calcined at 350 and 550 °C were prepared and characterized by TGA, XRD, BET, NH_3_‐TPD, TEM, H_2_‐TPR, SEM, EDX, XPS and DRIFT‐Pyridine techniques. The prepared catalysts were used in a fixed bed reactor under reaction conditions in which the temperature ranged from 180–300 °C with weight hourly space velocity (WHSV) = 12.1 h^‐1^. It was observed that all catalysts calcined at 550 °C (γ‐Al_2_O_3_ support phase) exhibited higher activity than those calcined at 350 °C (γ‐AlOOH), and this is due to the phase support change. Furthermore, the optimum Cu loading was found to be 6% Cu/γ‐Al_2_O_3_ with this catalyst also showing a high degree of stability under steady state conditions and this is attributed to the enhancement in surface acidity and hydrophobicity.

**CONCLUSION:**

The addition of copper to the support improved the catalyst properties and activity. For all the copper modified catalysts, the optimum catalyst with high degree of activity and stability was 6% copper loaded on gamma alumina. © 2017 The Authors. *Journal of Chemical Technology & Biotechnology* published by John Wiley & Sons Ltd on behalf of Society of Chemical Industry.

## INTRODUCTION

Recently, increasing attention has been directed towards the production of alternative, environmentally friendly fuels thereby decreasing the dependency on crude oil and affording associated improvements towards the mitigation of air pollution.^1^, ^2^ There are numerous cleaner fuel sources available such as biogas, hydrogen or, dimethyl ether (DME), with the latter being one of the most promising alternatives.^3^, ^4^ DME has been utilised in a broad range of applications and since the early 1990s has been considered as an alternative for diesel vehicles.^1^ In October 2014, Volvo made an announcement to modify its plan for North American alternative fuels by progressing the production of DME‐powered vehicles for the North American market.^5^

DME, having the simplest ether structure and the absence of a C–C bond, implies the generation of fewer undesired combustion by‐products such as hydrocarbons or particulates. As it is generally produced from clean gas streams (either directly or indirectly) it contains neither sulphur compounds nor aromatics and it is non‐carcinogenic, non‐teratogenic, non‐mutagenic and non‐toxic.^6^, ^7^

DME is produced by either indirect or direct synthesis. In the former method, methanol is dehydrated over a solid catalyst (reaction 1), while in the latter method, which is increasingly being employed, DME is produced from synthesis gas^8^, ^9^, ^10^, ^11^ over hybrid catalysts comprising a metal oxide to facilitate the synthesis of methanol and a solid acid catalyst for the methanol dehydration (MTD) reaction (reaction 2)^12^, ^13^

(1) $$2{\mathrm{CH}}_{3}\mathrm{OH}⇄\;{\mathrm{CH}}_{3}{\mathrm{OCH}}_{3}+H_{2}O$$

(2) $$3\mathrm{CO}+3H_{2}↔{\mathrm{CH}}_{3}{\mathrm{OCH}}_{3}+{\mathrm{CO}}_{2}$$

Of the two methods, DME is predominantly produced commercially via the catalytic dehydration of methanol (reaction 1). Solid acid catalysts^14^ such as γ‐Al_2_O_3_,^15^, ^16^, ^17^, ^18^ crystalline aluminosilicates,^19^ zeolites,^7^, ^12^, ^20^, ^21^, ^22^ and phosphates including aluminium phosphate^23^ are used, with γ‐Al_2_O_3_ and zeolites being the most common acid catalysts employed. It is evident that in almost all rate equations for dehydration the reaction rate is proportional to the square root of the methanol concentration. This indicates that the MTD reaction undergoes dissociative adsorption of methanol on the catalyst surface.^24^

Metal loaded aluminas can catalyse a wide range of reactions. Of interest here is the copper loaded versions which have been used in reactions as diverse as methanol synthesis,^25^ water gas shift,^26^ soot oxidation,^27^ biomass pyrolysis,^28^ methanol steam reforming^29^ and carbon monoxide (CO) oxidation.^30^ More specifically, mixed metal systems based on Cu/ZnO/Al_2_O_3_ with zeolites and other acid catalysts have been used in the direct production of DME from synthesis gas.^25^, ^31^, ^32^

Chattopadhyay *et al*.^28^ performed a thermogravimetric study on the pyrolysis of biomass with Cu/Al_2_O_3_ catalysts. They found that there was an increase in the catalytic activity with increasing Cu loading despite a decrease in the surface area and pore volume. Similarly, a study by Lopez‐Suarez *et al*.^27^ on soot oxidation using Cu/Al_2_O_3_ catalysts with metal loadings from 0.64 to 8.8 wt% demonstrated that the surface area decreased progressively with increasing copper, ranging from 88 m^2^ g^‐1^ for the bare support to 70 m^2^ g^‐1^ for the 8.8 wt% Cu/Al_2_O_3_ catalyst as expected. This result provides evidence that the support surface was partially blocked by Cu loading, which was further supported by Luo *et al*.^30^ who investigated CO oxidation and concluded that the formation of CuAl_2_O_4_ inhibits the CuO diffusion into the bulk Al_2_O_3_ support. In a recent study by Zhan *et al*.,^33^ they studied the effect of Cu loading on the acidity of mordenite zeolite by using FTIR analysis of adsorbed pyridine on the support. They found that after Cu loading, the Lewis acidic sites on the alumina support increased as copper cations act as electron acceptors which can also be considered as Lewis acids.^33^

In addition to the loading of Cu, the calcination temperature is also known to affect the catalytic activity in heterogeneous catalysis. For instance, Yahiro *et al*. studied the activity of copper supported on γ‐Al_2_O_3_ for the water–gas‐shift reaction.^26^ They reported that the BET surface area of Cu/Al_2_O_3_ gradually decreased with increasing calcination temperature with a corresponding increase in the catalyst bulk density.

For evaluating the catalytic performance, solid acid catalyst stability is also crucial. Yoo *et al*. have reported the production of DME from synthesis gas using Cu/ZnO/Al_2_O_3_ with various silico‐aluminophosphate catalysts^34^ and have concluded that the strength of the acid sites significantly affected the long‐term catalytic performance due to the formation of coke and subsequent catalyst deactivation. Clearly, strong acid sites, which are favourable for low‐temperature dehydration, tend to lead to coke formation or other undesired by‐products at high operating temperatures. Furthermore, in the MTD process water is produced which also has a significant effect on catalyst deactivation^14^, ^15^, ^23^, ^35^ as γ‐Al_2_O_3_ is hydrophilic,^36^ and facilitates strong water adsorption. Both water and methanol compete for adsorbing on the active sites of γ‐Al_2_O_3_ with water being adsorbed more strongly.^15^

It is possible to increase the hydrophobicity of the support thereby reducing the deactivation by water. Several techniques can be employed for this purpose; for instance, Reinosa *et al*.^37^ studied copper based hydrophobic ceramic nanocoatings where hydrophobic characteristics were measured using copper based glazes (5, 10, 15, 20 and 25 wt%) and from these results an optimum hydrophobicity (15 wt% Cu) was identified. They prepared a multifunctional ceramic coating for ceramic tiles applications. Dhere *et al*.^38^ enhanced the hydrophobicity of silica films using iron and copper metal acetylacetonate (acac) and heat treatment. The contact angles for water on the Cu (acac)_2_‐containing silica film and the heat‐treated film were 76° and 142°, respectively, permitting these materials to be categorised as hydrophobic or superhydrophobic. They prepared the hydrophobic materials in order to protect the solid surface due to the action of water adsorption.

The above discussion highlights the role of alumina in the dehydration reaction, the importance of acid site strength and the poisoning by either coke or water. Furthermore, it suggests that the addition of a metal onto the support could modify the properties of the catalyst surface, for instance, the addition of copper to the alumina surface could decrease the water adsorption by changing the hydrophobicity of the surface, leading to an increase in activity. However, high Cu loadings could partially block the pore volume of the alumina support. The balance between these two effects suggests that an optimum loading exists where it is recognised that this will likely be a function of the original support. In previous work^39^ a comparative study was performed between the two cheapest and most readily available alumina precursors, aluminium nitrate (AN) and aluminium chloride (AC). In this study, at all calcination temperatures, all η‐Al_2_O_3_ catalysts prepared from AN exhibited activity higher than γ‐Al_2_O_3_ catalysts that prepared from AC. Herein, we investigate the potential benefits from loading of Cu onto AC alumina for the production of DME via the dehydration of methanol. We report the activities of the catalyst at both low loadings (1, 2, 4 and 6%) and high loadings (10 and 15% wt/wt).

## MATERIALS AND METHODS

### Chemicals

The chemicals used in the present study were all of analytical grade and supplied by Aldrich, UK. These included aluminium chloride anhydride (AlCl_3_, 99%) ammonia solution (35%) and copper oxalate hemihydrate (CuC_2_O_4_.1/2H_2_O, 98%). The γ‐Al_2_O_3_ (BET = 117 m^2^ g^‐1^, pore size = 1.035 nm) was prepared by crushing γ‐Al_2_O_3_ pellets (Alfa Aesar). The He, H_2_ and air gases were purchased from BOC with purity 99.99%.

### Pure catalysts preparation

The preparation of the alumina support has been described elsewhere.^39^ It was prepared from aluminium chloride anhydride that was then precipitated by ammonia solution, the resulting precipitate was calcined at either 350 or 550 °C and designated as AC350 and AC550, respectively.

### Copper metal loading on pure catalysts preparation

Metal loaded catalysts were prepared by wet impregnation with the aid of sonication. Pure catalysts (AC350 (Boehmite)) and AC550 (γ‐Al_2_O_3_)) were loaded with x% (wt/wt) of copper (where x = 1, 2, 4, 6, 10 or 15%). Calculated amounts of copper oxalate hemihydrate were dissolved and/or dispersed with a known amount of support, in ∼5 mL deionized water. This was sonicated at 80 °C (Crest ultrasonic bath model 200 HT), at a frequency of 45 kHz, resulting in a homogeneous paste. All mixtures were then evaporated to dryness. Herein, catalysts loaded with 1, 2, 4, 6, 10, and 15% which were then calcined at 350 and 550 °C are denoted as follows: *X*% Cu/AC*Y* where *X* is the weight percentage of Cu and *Y* the calcination temperature.

### Catalyst characterization

Thermogravimetric analysis (TGA) was performed from ambient temperature to 600 °C at a heating rate of 10 °C per min, in a stream of dry N_2_ flowing at 40 cm^3^ min^‐1^, using a Perkin Elmer Thermogravimetric analyzer Pyris 1TGA. Changes in the sample mass were recorded during the temperature increase.

Powder X‐ray diffraction (XRD) analyses of the catalysts were carried out using a PANalytical X'Pert Pro X‐ray diffractometer. This diffractometer was equipped with a CuKα X‐ray source with a wavelength of 1.5405 Ǻ. Diffractograms were collected from 15° to 80°. The X‐ray tube was set at 40 kV and 40 mA. Once the scan had finished, the main peaks were selected and compared with diffraction patterns from the software library. The pattern with the highest percentage match was used. The particle size was calculated according to the Scherrer equation.

Brunauer–Emmett–Teller (BET) analysis was performed using a Micromeritics ASAP 2010 system. The BET surface areas and pore volumes were measured by N_2_ adsorption and the desorption isotherms at liquid nitrogen temperature (–196 °C).

The chlorine content was measured using oxygen flask analysis by subjecting the sample to combustion in an oxygenated flask containing water, followed by shaking; in which chlorine dissolved in water forming HCl solution is titrated with 0.02 mol L^‐1^ Hg_2_NO_3_ to obtain the percentage of chlorine.

The acidity of the catalysts was measured by the temperature programmed desorption of ammonia (NH_3_‐TPD). Before all experiments, the catalyst samples (50 mg) were treated *in situ* for 1 h under Ar at a flow rate of 50 cm^3^ per min, while the temperature was increased from ambient up to 300 °C at a heating rate of 15 °C min^‐1^, then the sample is cooled at 100 °C under Ar flow of 50 cm^3^ per min. The ammonia adsorption was performed under a stream containing a mixture of 1% NH_3_/Ar (50 cm^3^ min^‐1^) at 100 °C for 1 h. After the saturation of ammonia, the sample was purged with Ar for 0.5 h^40^ to remove weakly adsorbed NH_3_ on the catalyst surface. The ammonia desorption experiments were performed in the temperature range from 50 to 800 °C (heating rate 10 °C per min). The amount of NH_3_ desorbed from the catalyst was calculated by integration of the area under the desorption curve.

The relative strength of the Lewis acid sites was determined by diffuse reflectance infrared Fourier transform (DRIFT) analysis of adsorbed pyridine using a Bruker Vertex 70 FTIR Spectrometer equipped with a detector cooled with liquid N_2_. Before these measurements, samples were pre‐treated by outgassing at 120 °C for 0.5 h under an Ar atmosphere. Subsequently, the samples were saturated with pyridine at 50 °C then the physisorbed pyridine was removed by flushing at ∼25 °C with Ar gas for 0.5 h. Fresh samples (catalyst without pyridine) were used to record the IR background under Ar flow at 300 °C. Then, the pyridine (Py) adsorbed samples were placed in the DRIFT cell at 40 °C. The samples were heated under Ar at a flow rate of 50 cm^3^ min^‐1^ and the *in situ* DRIFT spectra were recorded at a resolution of 4 cm^‐1^ and with an accumulation of 56 scans every 30 s. The spectra after pyridine desorption were subtracted from those measured before pyridine adsorption (fresh samples) to observe the bands corresponding to Lewis and Brönsted acidic sites.

The static contact angle of the catalyst pellets with water was measured using a contact angle meter equipped with a CCD camera (FTA1000 Drop Shape Instrument ‐ B Frame system). The morphology of the catalysts surface was characterized by transmission electron microscopy (TEM) (Philips Tecnai F20 ST with high tension of 200 kV and a point resolution of 0.24 nm).

Temperature‐programmed reduction (TPR) was utilized to investigate the reducibility of the catalysts using a Micromeritics Autochem 2910 apparatus with the H_2_ uptake monitored by a thermal conductivity detector (TCD). For TPR measurements, the catalyst sample (0.1 g) was placed in a quartz tube and the temperature decreased to 0 °C under a flow of 15 mL pure Ar. The catalyst was then reduced by passing 5% H_2_/Ar at a flow rate of 30 mL min^‐1^ over the catalyst until a stable baseline was obtained after which the sample was heated at 10 °C per min up to 700 °C.

Scanning electron microscope (SEM) images were obtained on a FEI Quanta 250 FEG MKII with a high‐resolution environmental microscope (ESEM) using XT microscope Control software and equipped with an energy‐dispersive X‐ray (EDX) detector; a 10 mm^2^ SDD Detector‐x‐act (Oxford Instruments) utilising Aztec® EDS analysis software, was employed. Both systems used the same chamber.

XPS was performed in a PHI XPS Versaprobe 500 spectrometer (ULVAC–Physical Electronics, USA) with a quartz monochromator Al Kα radiation of energy 1486.6 eV. High‐resolution spectra of Cu*2p* were taken at a fixed pass energy of 20 eV, 0.05 eV step size and 100 ms dwell time per step. Surface charge was efficiently neutralised by flooding the sample surface with low energy electrons. Core level binding energies were corrected using the C 1 s peak at 284.8 eV as charge reference. For construction and fitting of synthetic peaks of high‐resolution spectra, mixed Gaussian‐Lorentzian functions with a Shirley‐type background subtraction were used.

### Catalyst activity

Catalyst activity tests were conducted in an isothermal fixed‐bed reactor made of stainless steel (6 mm OD). The catalyst bed consisted of 200 mg (250–425 µm) of a catalyst placed between two plugs of quartz wool. Aera mass flow controllers were used to control the flow of gases to the reactor. The liquid methanol was injected via a Cheminert® M Series liquid handling pump. A stable flow of methanol vapour to the reactor was established by passing the combined flow of He and methanol through a saturator system, with the evaporation chamber maintained at 150 °C. To prevent condensation, all lines were heated to 150 °C. This mixture was then fed to the fixed bed reactor. The reaction conditions used were 20% methanol under atmospheric pressure over a temperature range from 200 to 300 °C. The total flow rate was 100 cm^3^ min^‐1^. Before the reaction, the catalyst was activated in a stream of pure He at 325 °C for 0.5 h under atmospheric pressure and the catalyst was then reduced at 250 °C for 3 h with a 10% H_2_ in helium stream at a total flow rate of 50 cm^3^ min^‐1^. Then, the methanol and He mixture were fed to the reactor and samples analysed by on‐line gas chromatography (Perkin‐Elmer 500) equipped with a TCD and a flame ionisation detector (FID). A Hayesep DB column was used for the separation of CO, CO_2_, DME, MeOH, CH_4_, C_2_H_4_, C_2_H_6,_ ethanol, propanol, and butanol. Each data point was repeated five times to determine the reproducibility of the data for the products

As shown in Equation (3), the methanol conversion (*X*_*MeOH*_) was calculated on the basis of the molar flow rate of methanol in the feed (*F*_*MeOH, in*_) and in the outlet stream (*F*_*MeOH, out*_):

(3) $$X_{\text{MeOH}}=\frac{F_{\text{MeOH},\text{in}}-F_{\text{MeOH},\text{out}}}{F_{\text{MeOH},\text{in}}}$$

DME formation rate (*r*_*DME*_) was determined using Equation (4), which represents the actual moles of the product, DME, that are present in the reactor outlet stream per gram of the catalyst:

(4) $$r_{\mathrm{DME}}=\frac{F_{\mathrm{DME},\text{actual}}}{\mathrm{wt}.\text{of the catalyst}}\;\times 100\%$$

The selectivity for DME (*S*_*DME*_) was determined using Equation (5) as the ratio (expressed in mole%) between the content of carbon in the product DME and the sum of carbon content corresponding to all observed organic products which are present in the reactor outlet stream:

(5) $$S_{\mathrm{DME}}=\frac{2F_{\mathrm{DME}}}{F_{\mathrm{CO}2}+F_{\mathrm{CO}}+2F_{\mathrm{DME}}+\sum_{i}n_{\mathrm{Ci}}F_{i}}\;\times 100\%$$

Here, *F*_*DME*_, *F*_*CO2*_ and *F*_*CO*_ are the molar flow rates of DME, CO_2_ and CO, respectively, in the outlet stream, *n*_*Ci*_ is the number of carbon atoms for each of the hydrocarbons (by‐products) and *F*_*i*_ is the molar flow rate of these hydrocarbons.^25^

## RESULTS AND DISCUSSION

### Catalyst characterisation

Figure S1 and S2 (Supplementary information) show the XRD patterns of copper‐modified AC550 and AC350 and calcined at 300 °C, respectively; some copper oxalate was clearly observed at high Cu loadings indicating that a calcination temperature of 300 °C was insufficient to decompose all the copper oxalate to copper oxide. From Fig. (S1), three diffraction peaks were observed; CuO phase (2θ = 35, 38°), γ‐Al_2_O_3_ (2θ = 66, 45, 37°) and the remaining copper oxalate at 2θ = 22°. From Fig. (S2), three diffraction peaks were observed; CuO phase (2θ = 35.2, 38.5°), boehmite (2θ = 27, 37, 44, 48, 65°) and again the remaining copper oxalate at 2θ = 22°.

**Figure 1:**
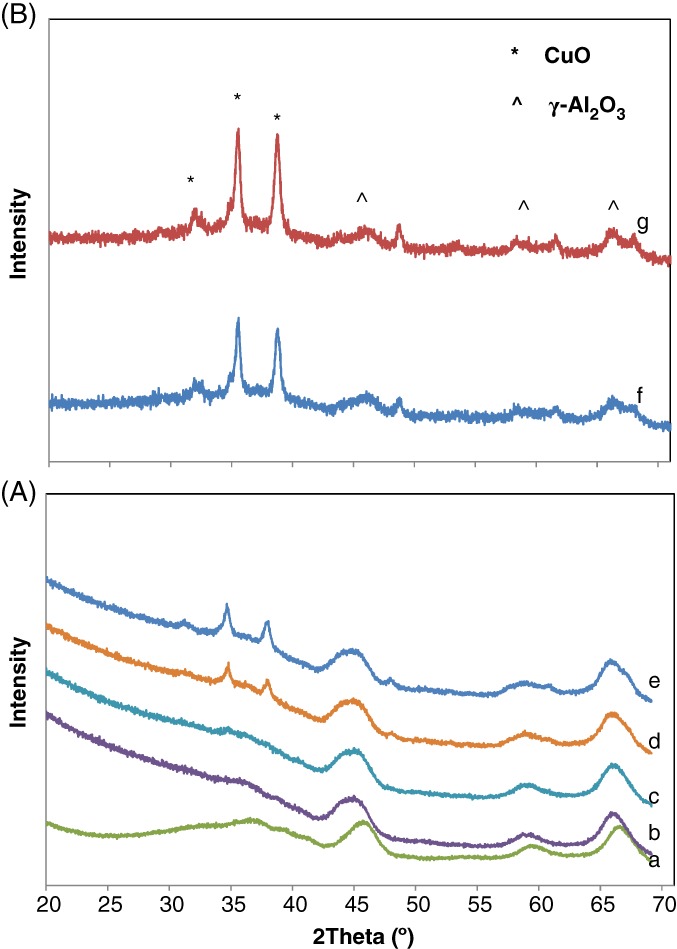
XRD patterns of copper modified AC550 catalyst calcined at 350 °C: (A) low copper loading (a) 0% (pure), (b) 1%, (c) 2%, (d) 4% and (e) 6%; (B) high copper loading, (f) 10% and (g) 15%.

**Figure 2:**
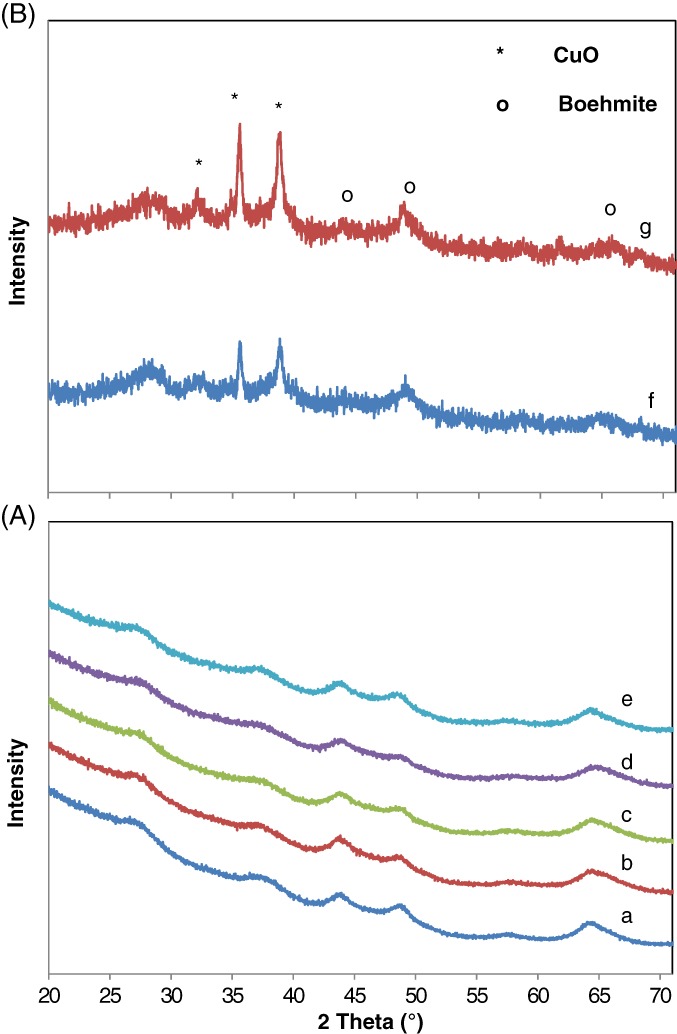
XRD patterns of copper modified AC350 catalyst calcined at 350 °C: (A) low copper loading (a) 0% (pure), (b) 1%, (c) 2%, (d) 4% and (e) 6%; (B) high copper loading, (f) 10% and (g) 15%.

To solve the problem of incomplete decomposition of copper oxalate, a higher calcination temperature of 350 °C was used which was sufficient to decompose all the copper oxalate into copper oxide as shown in Figs 1 and 2. In both figures, at low Cu loading (up to 2% for AC550 and 6% for AC350) no differences in diffraction lines were observed between the pure alumina catalysts and the modified catalysts, suggesting that copper is well dispersed on the alumina.^25^ However, with the increase in Cu loading, the copper oxide peak clearly appeared. Such results suggest the presence of a CuO phase (2θ = 35.2, 38.5°).

Copper‐loaded alumina catalysts were subjected to TGA analysis. Figure (S3) A displays the TGA curves of 1, 2, 4, 6, 10 and 15% Cu on alumina calcined at 550 °C. From the XRD results, the alumina support (calcined at 550 °C) is corresponded to the γ‐Al_2_O_3_ phase and this in line with a previous work.^39^ The total weight loss (%) calculated from ambient up to 600 °C was in the range 7.8–13.9%. As there was no observation of weight loss due to the phase change, it was attributed to the desorption of physisorbed water, carbon dioxide in the form of carbonate species^41^ and any remaining traces of chlorine within the bulk of the support. The chlorine content in AC550 and AC350 alumina supports was 0.38% and 0.8%, respectively, using the oxygen flask analysis described earlier. Within the region of desorption of water at 90 °C,^41^ the weight loss caused by the desorption of water was 2.06% and 3.1%, for a Cu loading of 6% and 15%, respectively. This showed that 6% Cu/AC550 was less hydrophilic than 15% Cu/AC550.

Figure S3 B shows the TGA curves for the same range of Cu loading on alumina which had been calcined at 350 °C (Boehmite). All samples showed three steps of weight loss corresponding to both the loss of free and physisorbed water and phase transformations to γ‐Al_2_O_3_. The first step started at 50 °C followed by two consecutive steps in the range 120–270 °C and 270–500 °C. After this weight loss, the rate of weight loss during heating up to 600 °C decreased with the formation of γ‐Al_2_O_3_. The total weight loss (%) calculated from ambient up to 600 °C ranged from 21% to 24%. Theoretically, the transformation of boehmite to γ‐Al_2_O_3_ should be associated with weight loss of 15%. The difference in weight loss of 6–9% can be attributed to the desorption of physisorbed water^41^ as well as above possible traces of chlorine remaining in the bulk of the pure alumina catalysts.

**Figure 3:**
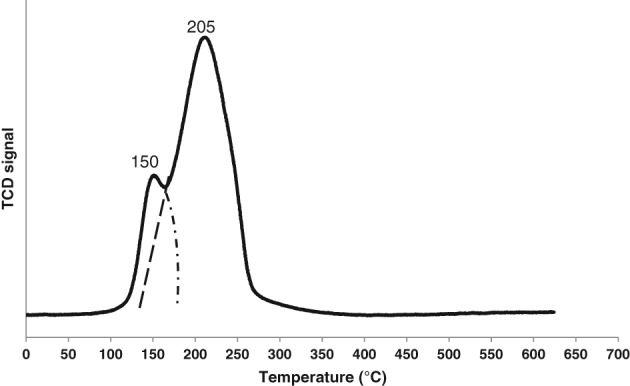
H_2_‐TPR profiles of 6%Cu /AC550.

H2‐TPR was used to investigate the types of CuO species formed on the surface of the alumina support. The H_2_‐TPR spectra of 6% Cu/AC550 in Fig. 3 shows two distinct reduction peaks meaning two apparent CuO species present on the surface of the catalyst, which is in agreement with the results reported by Aboul‐Fotouh *et al*. who showed ultrasonication for 1 h had a crucial effect on the surface morphology, where it produced two CuO species that were well dispersed and easily reducible and consequently, improved the catalytic production of DME.^42^

**Figure 4:**
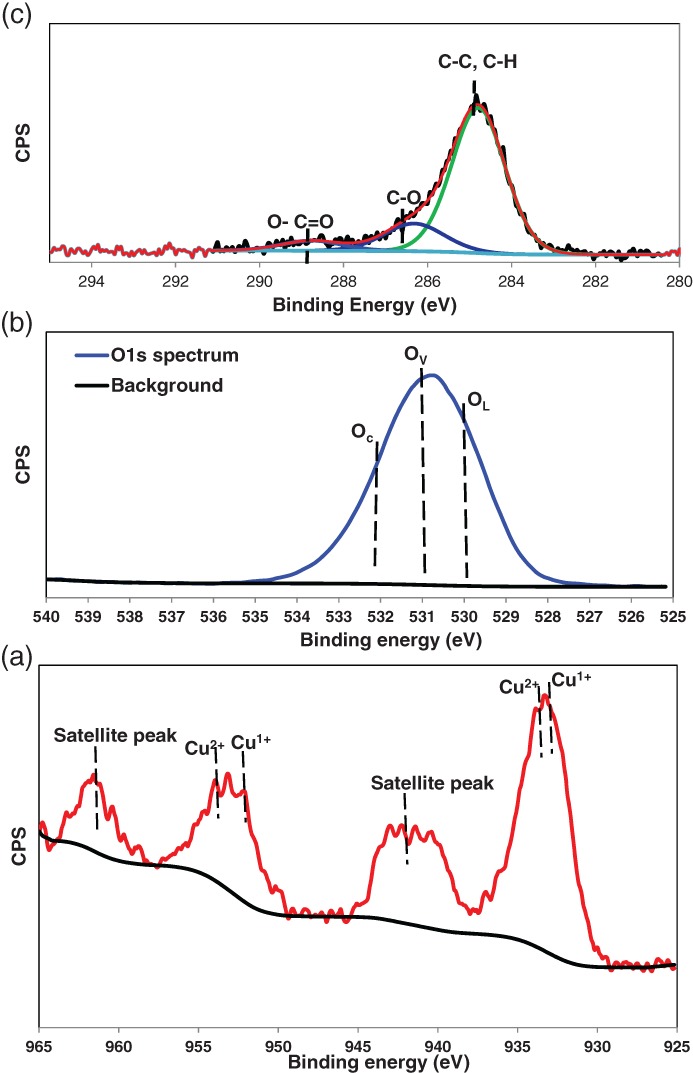
XPS of Cu2p (a), O1s (b) and C1s (c) for the 6% Cu/AC550 catalyst.

XPS was used to determine the oxidation states of copper on the surface of alumina support.The XPS analysis for 6%Cu /AC550 catalyst in Fig. 4 shows the copper *2p* region of the spectra (a). There was no indication of the presence of any Cu^0^ and copper was predominantly present as Cu^2+^ along with small peaks for Cu^1+^, which appeared with a binding energy of approximately 932.6 eV (Cu*2p*3/2) and 952 (Cu*2p*1/2) eV, however, Cu^2+^ appeared with a binding energy of approximately 933.2 eV (Cu*2p*3/2) and 953 (Cu*2p*1/2) eV.^43^, ^44^ This is in agreement with the TPR results as shown in Fig. 3 that showed there were two different types of copper oxides on the surface of the 6% Cu /AC550 catalyst. Furthermore, Fig. 4(b) shows the O1s region spectra in order to investigate the oxygen species on the surface of 6%Cu/AC550 catalyst. It is apparent that oxygen exhibits an oxidation state of O^2‐^, existing as oxygen lattice, vacancies and oxygen dissociated with binding energies at O_L_ (530 eV), O_V_ (531 eV) and O_C_ (532 eV), respectively.^45^ XPS results showed the existence of CuO and Cu_2_O phases on the surface of alumina support. Since the 6%Cu/AC550 catalyst was left in the air, so the adventitious carbon is expected to present in the form of adsorbed carbonaceous species (CO, CO_2_ and CO_3_) with adventitious carbon C–C at 284.8 eV, adsorbed CO/CO_2_ (C–O at 286.2 eV) and O–C = O at ∼288.6 eV (Fig. 4(c))^46^ and this is in agreement with the TGA results that showed weight loss at around 300 °C.

Figure (S4) shows a typical N_2_ adsorption isotherm of a modified catalyst calcined at 550 °C. The isotherm exhibited a hysteresis typical of type IV behaviour, which is characteristic of mesoporous solids.^47^ Surface area and pore volume results of all catalysts are given in Table 1. The adsorption volume of all catalysts calcined at 550 °C was significantly greater than those calcined at 350 °C. It is obvious that the surface area value and pore volume increased initially at a Cu loading of 1% and then decreased with increase in the Cu loading; this may be due to the opening of new active sites at low loading of Cu (1% Cu), in agreement with the recent work done by Braga *et al*.,^48^ then followed by pore blocking at high loadings of copper (2, 4, 6, 10 and 15% Cu) and consequently a decrease in the surface area.^28^ Comparison of the isotherms of the supports and metal loaded catalysts revealed no considerable shift in the reduced pressure, P/P_o_, range (not shown). For the modified samples with low Cu loading, hysteresis was observed, but at higher Cu loading, only a slight shift was observed. This indicates that the pore diameters of the pure alumina (AC350 and AC550) did not significantly change with lower loading, but started to decrease with higher Cu loading (see Table 1).

**Table 1:** Surface area and particle sizes along with catalytic activity of the different phases of pure and modified alumina catalysts

Catalyst abbreviation	Surface area		Particle size (nm)	Selectivity (%^a^)	Acid density(µmoles NH_3_ m^‐2^)	DME formation rate, (mmol h^‐1^ g^‐1^)
	S_BET_ (m^2^ g^‐1^)	Pore volume (cm^3^ g^‐1^)				
AC350	300	0.22	3.1	100	85.04	33.69
1% Cu/AC350	372	0.28	3.5	100	67.63	34.36
2% Cu/AC350	340	0.25	3.9	100	85.11	36.51
4% Cu/AC350	322	0.23	4.1	100	91.67	42.31
6% Cu/AC350	298	0.22	6.5	100	115.98	50.68
10% Cu/AC350	254	0.19	10.9	71.2	111.72	44.56
15% Cu/AC350	223	0.14	16.1	63.9	105.58	38.01
AC550	278	0.35	3.7	100	40.90	83.32
1% Cu/AC550	283	0.35	4	100	43.31	84.83
2% Cu/AC550	273	0.34	4.2	100	45.14	85.36
4% Cu/AC550	265	0.33	4.3	100	48.99	89.66
6% Cu/AC550	257	0.33	4.6	100	54.79	92.87
10% Cu/AC550	234	0.29	5.0	95.1	51.83	91.05
15% Cu/AC550	229	0.17	5.9	92.97	38.27	85.90

a T = 250 °C; He flow rate = 80 mL min^‐1^; WHSV: 12.1 h^‐1^.

**Figure 5:**
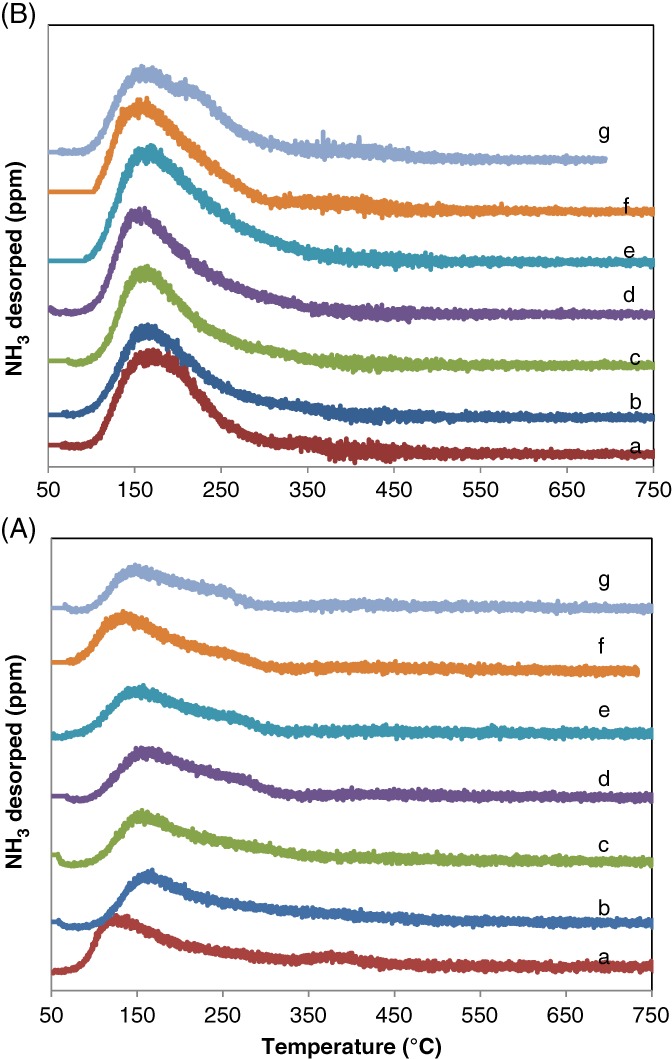
NH_3_‐TPD diagram. (A) AC550 with different copper loadings (a) 0%, (b) 1%, (c) 2%, (d) 4%, (e) 6%, (f) 10% and (g) 15%; (B) AC350 with different copper loadings (a) 0%, (b) 1%, (c) 2%, (d) 4%, (e) 6%, (f) 10% and (g) 15%.

The surface acidic properties of AC550 and AC350 samples with various Cu loadings were determined by NH_3_‐TPD. From Fig. 5, a peak maximum appeared in every TPD profile. Figure 5(A) shows the NH_3_‐TPD profile of AC550 (pure supports with 0% loading); a broad peak with maximum at approximately 120 °C was observed and for AC350 (Fig. 5(B)) this maximum was observed at about 150 °C. After modification with copper the peak maximum was shifted to higher temperatures up to 170 °C for AC 350 and AC 550, respectively, at a Cu loading of 6%, and then decreased with further Cu loading with the development of small shoulders for Cu loading of 10% and 15% indicating that the acid strength of the catalyst increased with the addition of copper up to 6% with a constant acid number (see Table 1).^33^

**Figure 6:**
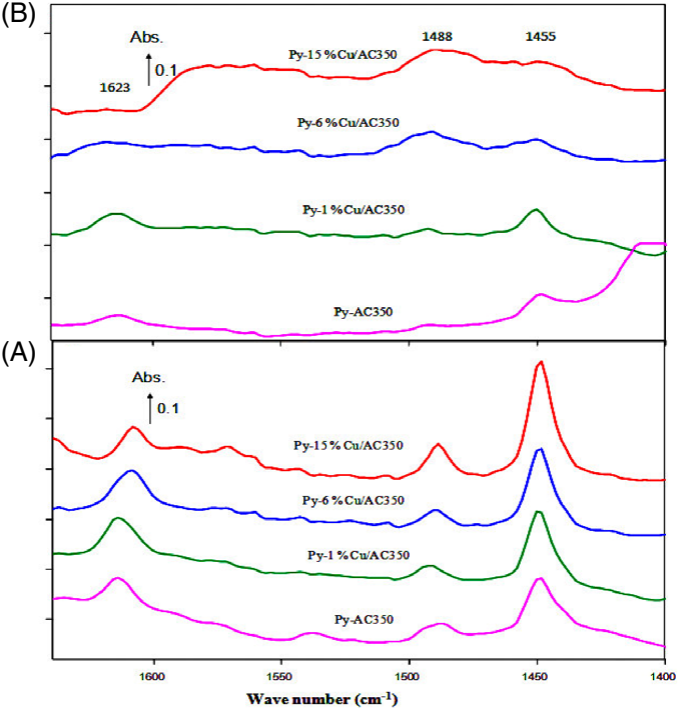
In situ DRIFTS spectra of pyridine adsorption pyridine adsorbed on AC350 catalysts following thermal treatment (A) at 200 °C and (B) at 300 °C in the region 1600–1400 cm^‐1^_._

To differentiate between Lewis and Bronsted acid sites and to determine their strength DRIFT‐Pyridine experiments were performed. Figure 6 shows the infrared spectra of the pyridine adsorbed on AC350 catalysts with Cu loadings between 0% and 15%.

**Figure 7:**
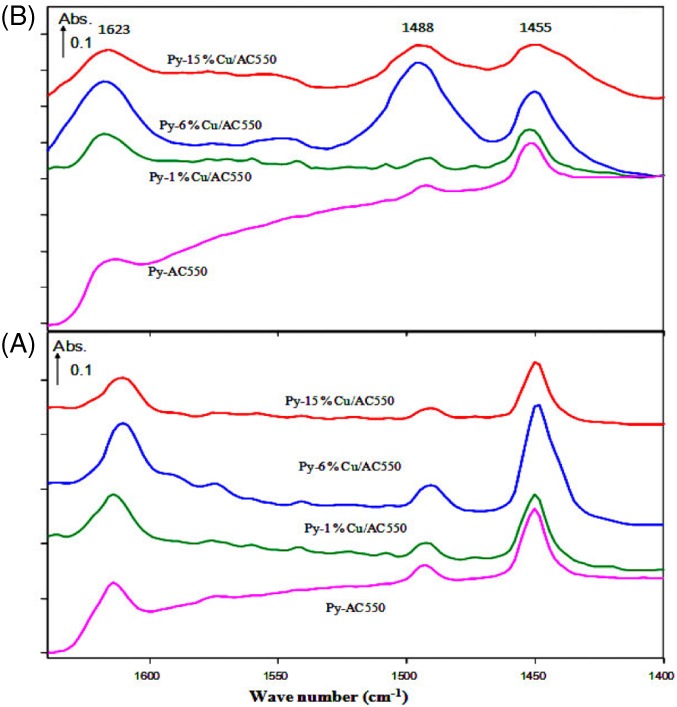
In situ DRIFTS spectra of pyridine adsorption. Pyridine adsorbed on AC550 catalysts following thermal treatment (A) at 200 °C and (B) at 300 °C in the region 1600–1400 cm^‐1^_._

Figure 7 shows the corresponding spectra for the AC550 catalysts. The pure and the modified catalysts were compared by thermal treatment at 200 and 300 °C in the region of 1600–1400 cm^‐1^_._ As shown in the spectrum (A) of Fig. 7, absorbance bands were observed at 1455, 1488, and 1623 cm^‐1^. The band observed at 1455 cm^‐1^ is attributed to the presence of hydrogen‐bonded pyridine adsorbed on Lewis acid sites.^49^, ^50^ Bands observed at 1623 and 1455 cm^‐1^ are attributed to Strong Lewis bound pyridine and those observed at 1575 cm^‐1^are attributed to weak Lewis bound pyridine.^50^ The band observed at about 1488 cm^‐1^ is attributed to pyridine adsorbed on both Lewis and Brönsted acid sites. From spectra (A), all catalysts exhibited bands at 1623 and approximately 1455 cm^‐1^, corresponding to strong Lewis acidic sites and another small band at 1488 cm^‐1^, attributed to both Lewis and Brönsted sites. From the DRIFTs spectra that the Lewis acidic sites were clearly responsible for the acidity in the pure and the modified catalysts, which are in agreement with the literature and previous work.^39^, ^51^ From spectra (A) Fig. 7, there was no significant change observed on either the type or strength of the acidic sites of the pure and the modified catalysts. Notably, at 300 °C (spectra (B)) almost all the peaks disappeared with the thermal treatment except for those corresponding to 6%Cu/AC550, where the peak at 1488 cm^‐1^ increased thus indicating stronger acidic sites.

**Figure 8:**
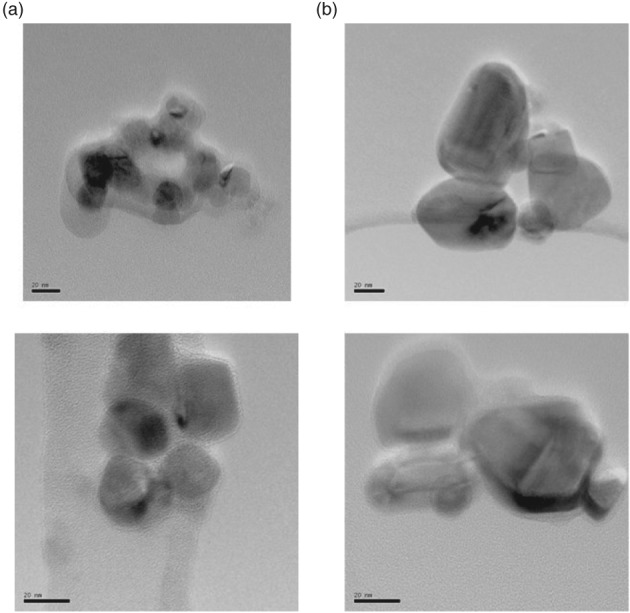
Representative TEM micrographs of (a) 6% Cu/AC550 and (b) 15% Cu/AC550.

From the TEM images shown in Fig. 8, different‐shaped copper particles were observed. From these images, the particles in the 6% Cu/AC550 were clearly spherical and they became more angular with higher loadings of Cu. In addition, the particle size started to increase with the higher loading as confirmed from the XRD results.

**Figure 9:**
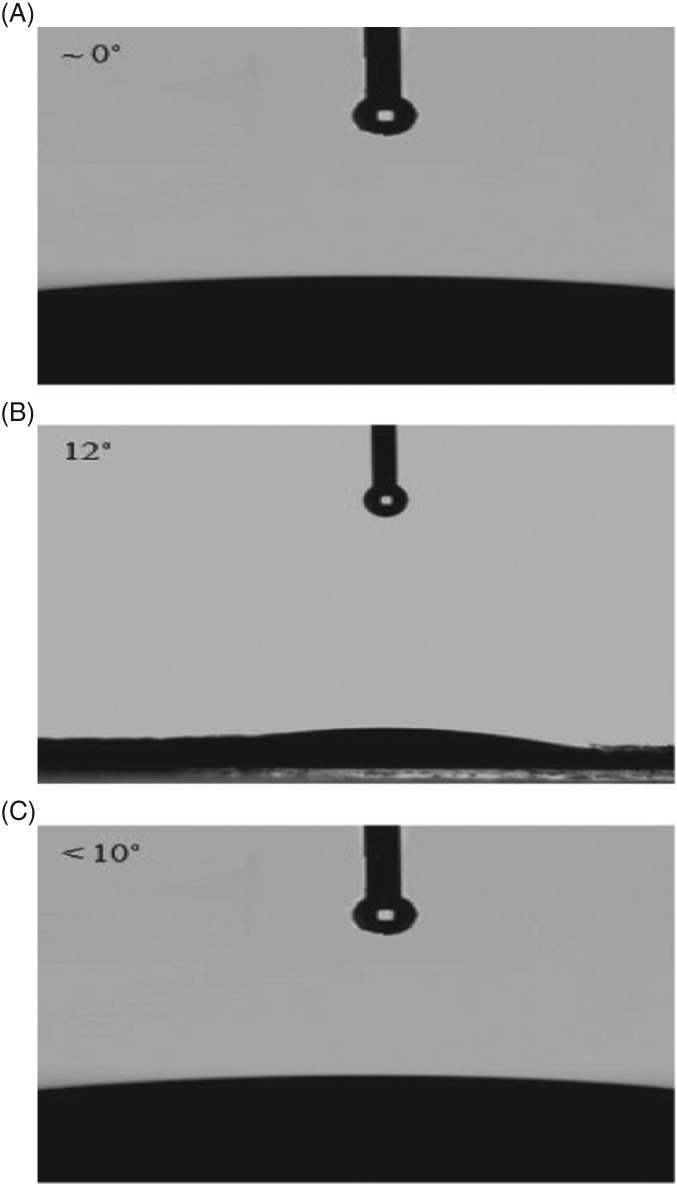
Comparison between hydrophilicity of catalyst surface of (A) AC550, (B) 6% Cu/AC550 and (C) 15% Cu/AC550.

Based on the previous discussion, the hydrophobicity of the catalyst may change with increasing Cu loading. One simple method of testing this hypothesis is to examine the contact angle of water on pellets made from the respective catalyst powders. As shown in Fig. 9, the contact angle for the AC550 changed with Cu loading. For the pure support, the contact angle was ∼0°; however, it increased to 12° with increasing Cu loading to 6%. However, with further increase of Cu loading, the contact angle decreased again to less than 10°. These results suggest that the surface changed from being superhydrophilic to hydrophilic and then back again to superhydrophilic with increase in the Cu loading. This result is in agreement with TGA measurements in Fig. S3 A, as in the temperature range of water desorption (100–200 °C), 6% Cu/AC550 showed the lowest percentage weight loss, indicating its hydrophobic character.

Schematic representation 1 (Fig. S5) shows that at low Cu loading, the water adsorbed easily on the surface of alumina catalyst, while at 6% Cu loading there was a uniform dispersion of copper that enhanced the hydrophobicity. However, at higher Cu loading (15%), copper clusters were formed and water adsorbed between these clusters and consequently decreased the hydrophobicity.

Figure S6 demonstrates the EDX results for the 2%, 6% and 15% Cu/AC550. At a high loading of Cu on the surface of the alumina support, the dispersion of Cu apparently decreased and started to form a cluster of copper particles which is also in agreement with the SEM images (Fig. S7). From the SEM images, with increase in Cu loadings, the shape of the particles apparently changed and with high loading (15% Cu) the particles formed clusters and big particles which confirmed the BET results: with increase in Cu loading, as the surface area dramatically decreased, copper started to block the pore surface of the alumina (AC550) support.

### Catalyst activity

**Figure 10:**
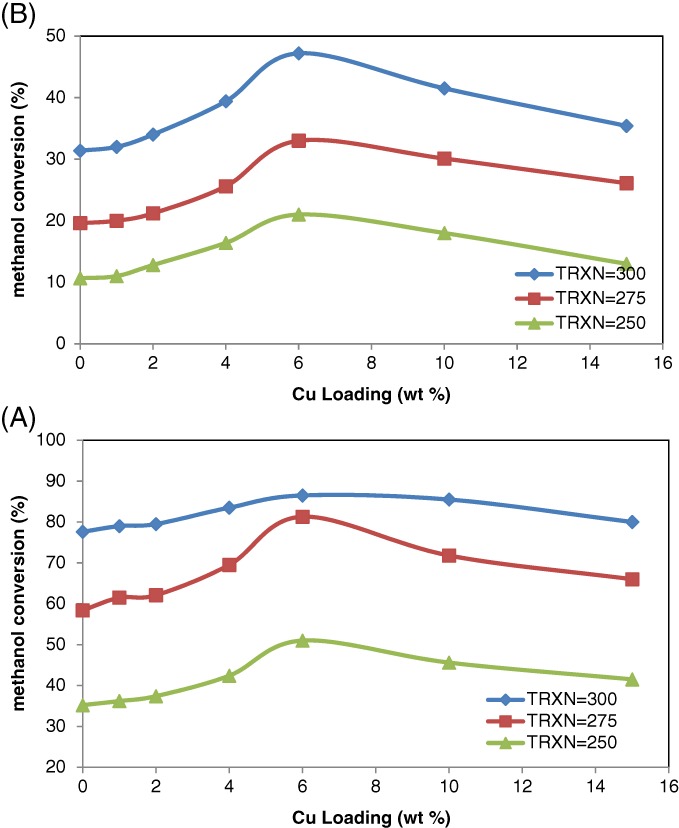
Effect of copper loading on methanol conversion over catalysts: (A) AC550 catalysts; (B) AC350; at different reaction temperatures (T = 250–300 °C; catalyst weight = 200 mg; He flow rate = 80 mL min^‐1^; WHSV: 12.1 h^‐1^).

The effects of Cu loading on AC350 and AC550 catalysts for reactions carried out over the temperature range 250–300 °C are shown in Fig. 10. From Fig. 10(A) and (B) for each reaction temperature, the methanol conversion clearly increased with increase in the Cu loading until it reached maximum at a Cu loading of 6%, whereafter, it declined with further increase in Cu loading. For instance, at a reaction temperature of 300 °C, the conversion of methanol over AC350 was 31.38%, which increased to 47.2% over 6% Cu/AC350 and then decreased to 35.4% at 15% Cu loading.

**Figure 11:**
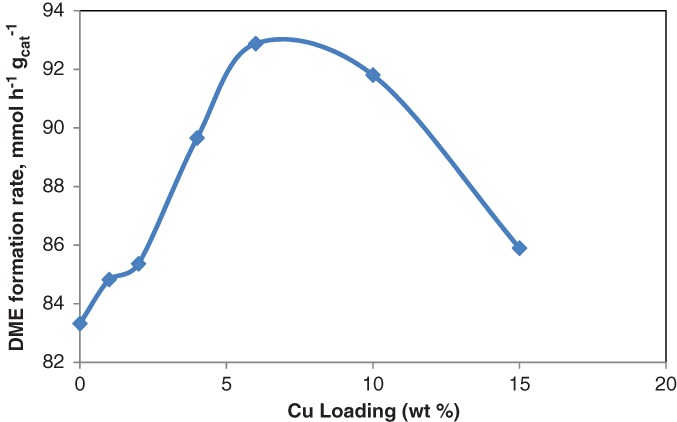
Effect of copper loading on the rate of DME formation over AC550 catalyst at T = 300 °C; catalyst weight = 200 mg; He flow rate = 80 mL min^‐1^; WHSV: 12.1 h^‐1^.

The same result can be observed from the DME reaction rate shown in Fig. 11. Such differences are attributed to differences in the surface acid strength as well as hydrophobicity properties. As discussed above for the 6% Cu loading, the hydrophobicity of the surface improved from superhydrophilic to hydrophilic (see Fig. 9) and stronger acidic sites were observed (Fig. 7 spectra (B). The DME reaction rate was a function of Cu loading as shown in Fig. 11, showing that 6% Cu/AC550 catalyst exhibited the highest DME reaction rate.

**Figure 12:**
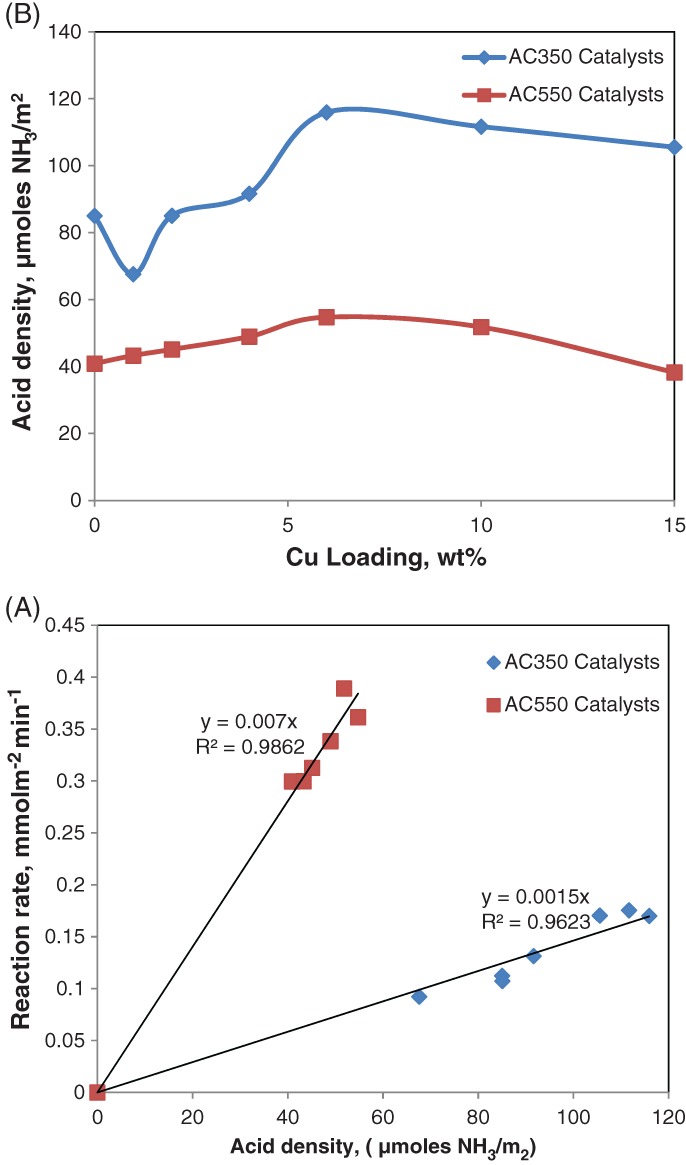
(A) Effect of acid density on DME reaction rate. (B) Effect of copper loading on the acid density over AC550 and AC350 catalysts; reaction temperature T = 300 °C; catalyst weight = 200 mg; He flow rate = 80 mL min^−1^; WHSV: 12.1 h^−1^.

Figure 12(A), shows the effect of acid density on the DME reaction rate and Fig. 12(B) shows the effect of Cu loading on the acid density over AC550 and AC350 catalysts; at a reaction temperature of 300 °C, catalyst weight of 200 mg, He flow rate of 80 mL min^‐1^ and weight hourly space velocity (WHSV) of 12.1 h^‐1^. With increase in the Cu loadings, the acidity clearly increased and with increasing acid density the reaction rate increased.^33^

**Figure 13:**
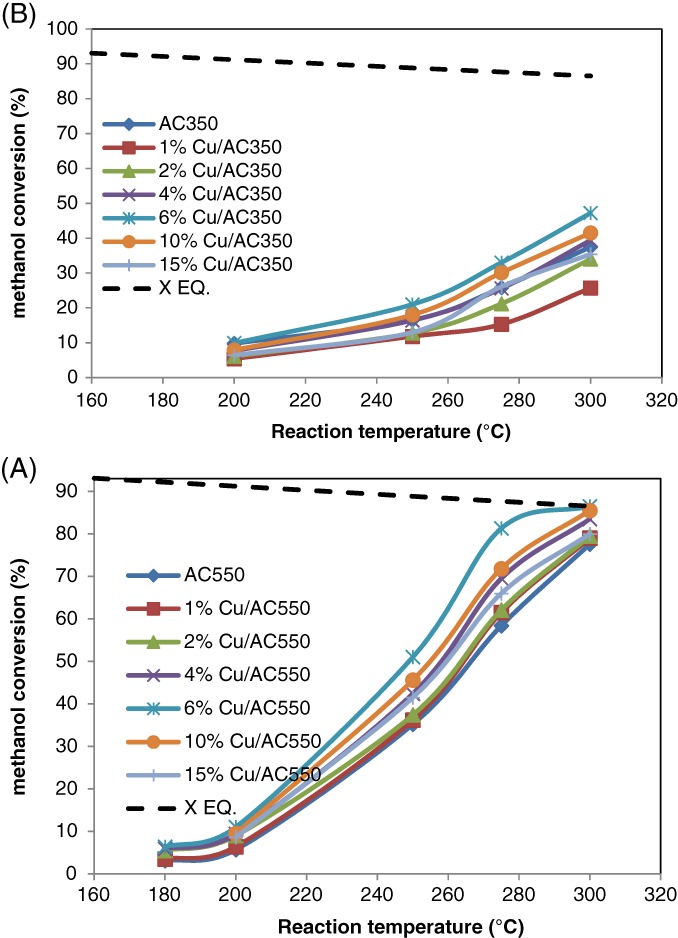
Effect of reaction temperature on methanol dehydration to DME with different copper loadings: (A) AC550 catalysts and (B) AC350. (T = 180–300 °C; catalyst weight = 200 mg; He flow rate = 80 mL min^‐1^; WHSV: 12.1 h^‐1^.)

Figure 13 shows the effect of reaction temperature; the conversion increased significantly with increasing reaction temperature. From this result, after modification with copper, the conversion approached the equilibrium curve while in Fig. 13(b) this did not occur. These results can be attributed to the phase of the support, in that the samples in Fig. 13(A) were calcined at 550 °C, i.e. γ‐Al_2_O_3_ phase, while those in Fig. 13(B) represented the lower activity Boehmite phase. The thermodynamic equilibrium conversion for 6% Cu/AC550 and 10% Cu/AC550 was obtained at temperatures greater than 300 °C. Given the equilibrium constraints, the conversion of methanol did not exceed 86.5%; hence the maximum conversion of methanol is limited under the reaction conditions (300 °C) employed. Each point in Fig. 13 was repeated five times to ensure the reproducibility of the data. To examine the effects of mass transfer limitations, reactions were carried out over the most active catalyst, 6% Cu/AC550 using 250–425 µm pellets or a fine powder. The percentage conversion of methanol over the different sizes of catalyst particles at different temperatures is shown in Figure S8. The catalytic activities were identical, i.e. the reaction is not controlled by the particle size of the catalyst and, consequently, is not limited by diffusion through the catalyst pellet.

**Figure 14:**
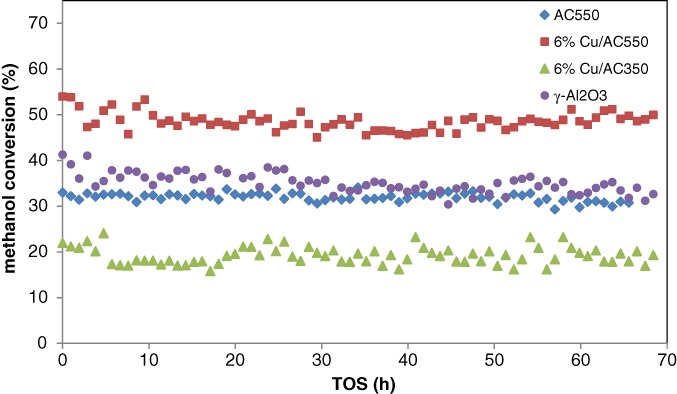
MeOH conversion with time on stream over AC550, 6% Cu/AC550, 6% Cu/AC350 and γ‐Al_2_O_3_. (T = 250 °C; catalyst weight = 200 mg; He flow rate = 80 mL min^‐1^; WHSV: 12.1 h^‐1^.)

Figure 14 shows the results of stability tests over a period of 70 h for the AC550 and the optimum modified catalysts calcined at 550 °C (6% Cu/AC550) and 350 (6%Cu/AC350) as well as those for commercially available γ‐Al_2_O_3_. Stability is one of the main requirements for such catalysts and from this figure, while all catalysts were reasonably stable, the optimum catalyst in terms of both activity and stability appeared to be 6% Cu/AC550 catalyst. In addition, the conversion of AC550 at 250 °C was clearly around 35.2% while for γ‐Al_2_O_3_, the conversion was around 37%. In our recent work, we proposed a novel green preparation route to prepare nano‐mesoporous γ‐Al_2_O_3_ from aluminium foil waste (AFW). Egypt has the largest aluminium company in the Middle East producing aluminium solid waste (ASW) which is mainly AC type, so our future work is to study the effect of Cu loading on AFW or ASW along with the kinetic modeling for the most active catalyst in this study (6% Cu/AC550).

## CONCLUSION

Herein copper loaded on alumina supports calcined at different temperatures was investigated for the production of DME from methanol. Two ranges of copper were loaded on these supports; low loading ranging from 1 to 6% wt/wt and high loading at 10 and 15% Cu wt/wt. Under the reaction conditions used in which the temperature ranged from 180 to 300 °C with a WHSV of 12.1 h^‐1^, all the catalysts calcined at 550 °C exhibited activity higher than those calcined at 350 °C. The addition of copper to the support improved the catalytic activity and within this study, the optimum catalyst was 6% Cu/AC550, attributed to the fact that the alumina support is partially blocked (as confirmed with TEM) at high Cu loading. The results also showed that the Cu dispersion decreased with increasing Cu loading, which resulted in secondary improvement of the bulk surface properties by changing the surface from superhydrophilic to hydrophilic at 6% Cu loading. Furthermore, this catalyst exhibited a high degree of stability and greater than 50% increase in conversion over the pure catalyst (AC550) as well as double the activity of that of the optimum catalyst calcined at 350 °C (6% Cu/AC350).

## Supporting information


**Figure S1.** XRD patterns of AC550 calcined at 300 °C A) low copper loading a) 0% (pure), b) 1%, c) 2%, d) 4% and e) 6%; B): high copper loading, f) 10% and g) 15%.
**Figure S2.** XRD patterns of AC300 calcined at 300 °C, A) low copper loading a) 0% (pure), b) 1%, c) 2%, d) 4% and e) 6%; B): high copper loading, f) 10% and g) 15%.
**Figure S3.** TGA curves for the catalysts in a N_2_ atmosphere with a heating rate of 10 °C/min, A): AC550 at different copper loadings, a) 1%, b) 2%, c) 4%, d) 6%, e) 10% and f) 15%.; B): AC350 at different copper loading, a) 1%, b) 2%, c) 4%, d) 6%, e) 10% and f) 15%.
**Figure S4.** N_2_‐Adsorption/desorption isotherm of 6% Cu/AC550.
**Figure S5.** Schematic representation 1: links the water adsorption at the surface of the catalyst with the copper dispersion at different Cu loadings on AC550.
**Figure S6.** EDX results of (A) 2% Cu/AC550, (B) 6% Cu/AC550 and (C) 15% Cu/AC550.
**Figure S7.** SEM images of (A) 2% Cu/AC550, (B) 6% Cu/AC550 and (C) 15% Cu/AC550 with magnification 1000 (left), 10000 (middle) and 20000 (right), respectively.
**Figure S8.** The effect of different particle sizes on the catalytic activity of 6% Cu/AC550, 250‐425 µm pellets (solid line) and powdered form (dash line). Reaction conditions: T = 180‐300 °C; catalyst weight = 200 mg; He flow rate = 80 ml/min; WHSV: 12.1 h^−1^.
