# Supplementary material for: Surface hydrophobicity and acidity effect on alumina catalyst in catalytic methanol dehydration reaction
Source: J Chem Technol Biotechnol. 2017 Sep 8;92(12):2952–62. doi: 10.1002/jctb.5371 (PMC5698741; doi:10.1002/jctb.5371)
Supplement: Supplementary file 1 — Figure S1. XRD patterns of AC550 calcined at 300 °C A) low copper loading a) 0% (pure), b) 1%, c) 2%, d) 4% and e) 6%; B): high copper loading, f) 10% and g) 15%. Figure S2. XRD patterns of AC300 calcined at 300 °C, A) low copper loading a) 0% (pure), b) 1%, c) 2%, d) 4% and e) 6%; B): high copper loading, f) 10% and g) 15%. Figure S3. TGA curves for the catalysts in a N2 atmosphere with a heating rate of 10 °C/min, A): AC550 at different copper loadings, a) 1%, b) 2%, c) 4%, d) 6%, e) 10% and f) 15%.; B): AC350 at different copper loading, a) 1%, b) 2%, c) 4%, d) 6%, e) 10% and f) 15%. Figure S4. N2‐Adsorption/desorption isotherm of 6% Cu/AC550. Figure S5. Schematic representation 1: links the water adsorption at the surface of the catalyst with the copper dispersion at different Cu loadings on AC550. Figure S6. EDX results of (A) 2% Cu/AC550, (B) 6% Cu/AC550 and (C) 15% Cu/AC550. Figure S7. SEM images of (A) 2% Cu/AC550, (B) 6% Cu/AC550 and (C) 15% Cu/AC550 with magnification 1000 (left), 10000 (middle) and 20000 (right), respectively. Figure S8. The effect of different particle sizes on the catalytic activity of 6% Cu/AC550, 250‐425 µm pellets (solid line) and powdered form (dash line). Reaction conditions: T = 180‐300 °C; catalyst weight = 200 mg; He flow rate = 80 ml/min; WHSV: 12.1 h−1. [file JCTB-92-2952-s001.docx]

Electronic Supplementary Information

For

**Surface Hydrophobicity and Acidity Effect on Alumina Catalyst in Catalytic Methanol Dehydration Reaction**

Ahmed I. Osman ^a,b*^, Jehad K. Abu-Dahrieh^a*^, David W. Rooney^a*^, Jillian Thompson^a^, Samih A. Halawy^b^, Mohamed A. Mohamed^b^

^a^CenTACat, Queen’s University Belfast, Belfast BT9 5AG, Northern Ireland, UK

^b^Chemistry Department, Faculty of Science - Qena, South Valley University,

Qena 83523 – Egypt

* Corresponding author: Jehad Abu-Dahrieh, David Rooney, Ahmed Osman

E-mail: [j.abudahrieh@qub.ac.uk](mailto:j.abudahrieh@qub.ac.uk), [d.rooney@qub.ac.uk](mailto:d.rooney@qub.ac.uk), aosmanahmed01@qub.ac.uk

Address: School of Chemistry and Chemical Engineering, Queen's University Belfast, David Keir Building, Stranmillis Road, Belfast BT9 5AG, Northern Ireland, United Kingdom

Fax: +44 2890 97 4687

Tel.: +44 2890 97 4269

**B)**

g

f

*** CuO**

**^o^ Cu oxalate**

**^ γ-Al_2_O_3_**

^o^

*

**A)**

d

c

e

b

a

**Figure (S1):** **XRD patterns of AC550 calcined at 300°C A) low copper loading a) 0% (pure), b) 1%, c) 2%, d) 4% and e) 6%; B): high copper loading, f) 10% and g) 15%.**

**B)**

o

o

o

*

*

*

g

f

*** CuO**

**o Boehmite**

**A)**

c

b

d

e

a

**Figure (S2):** **XRD patterns of AC300 calcined at 300°C, A) low copper loading a) 0% (pure), b) 1%, c) 2%, d) 4% and e) 6%; B): high copper loading, f) 10% and g) 15%.**

**B)**

c

d

e

f

b

a

**A)**

a

c

b

d

e

f

**B)**

**Figure (S3):** TGA curves for the catalysts in a N_2_ atmosphere with a heating rate of 10 °C/min, A): AC550 at different copper loadings, a) 1%, b) 2%, c) 4%, d) 6%, e) 10% and f) 15%.; B): AC350 at different copper loading, a) 1%, b) 2%, c) 4%, d) 6%, e) 10% and f) 15%.

**Figure S4:** N_2_-Adsorption/desorption isotherm of 6% Cu/AC550.


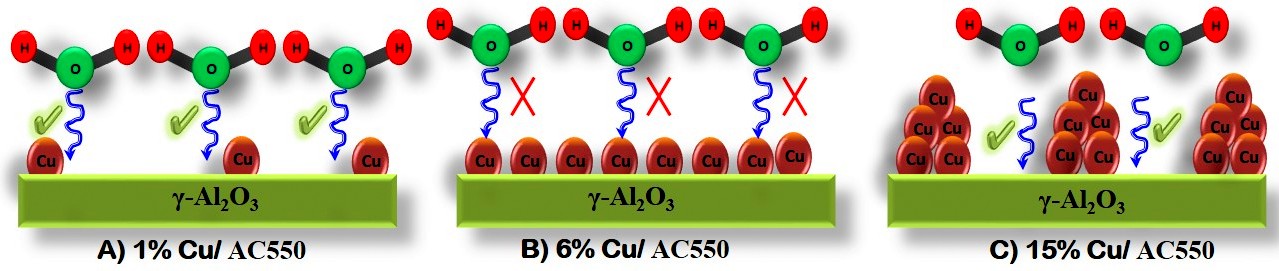


**Figure (S5)**: **Schematic representation 1:** links the water adsorption at the surface of the catalyst with the copper dispersion at different Cu loadings on AC550.


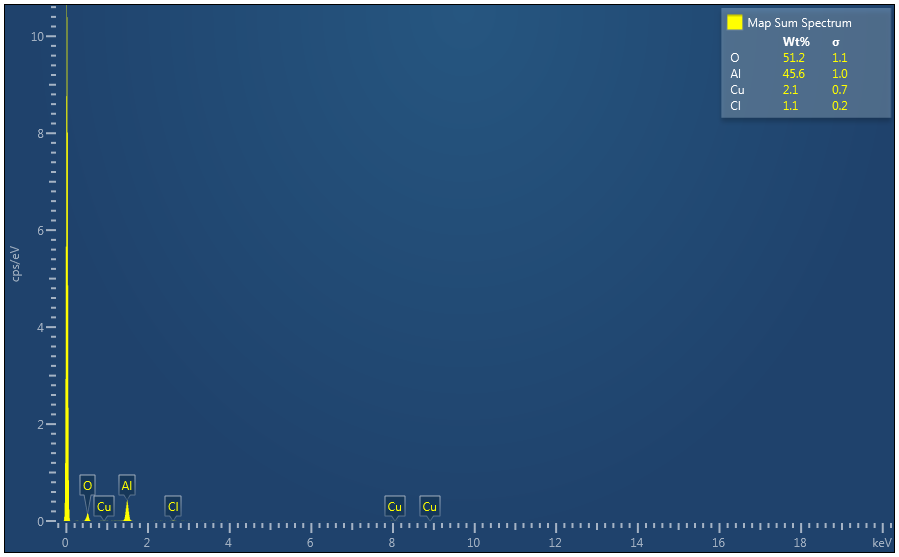

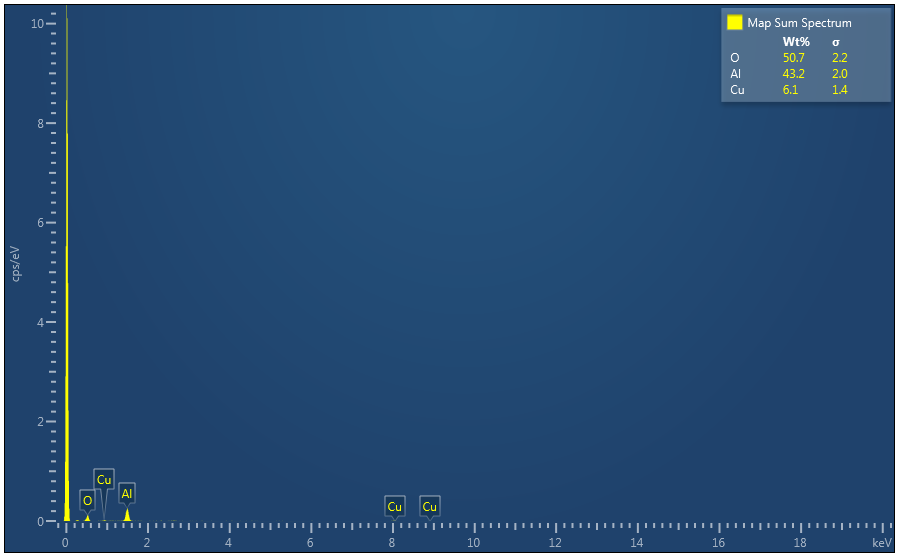

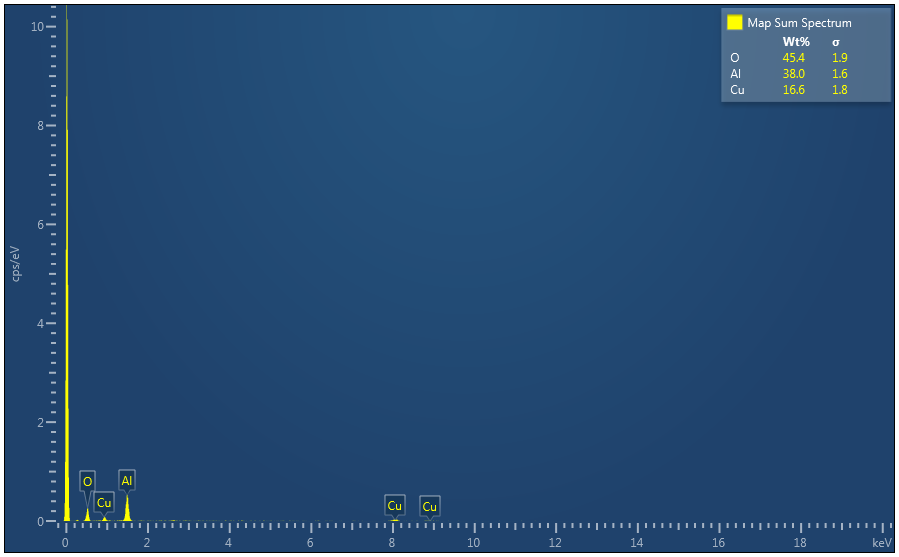


**C**

**B**

**A**

**Figure (S6)**: EDX results of (A) 2% Cu/AC550, (B) 6% Cu/AC550 and (C) 15% Cu/AC550.


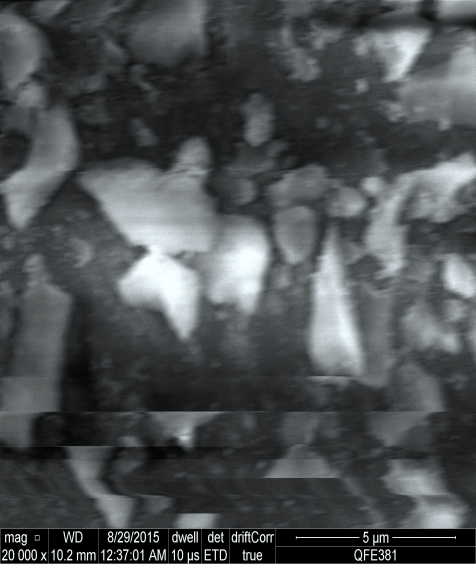

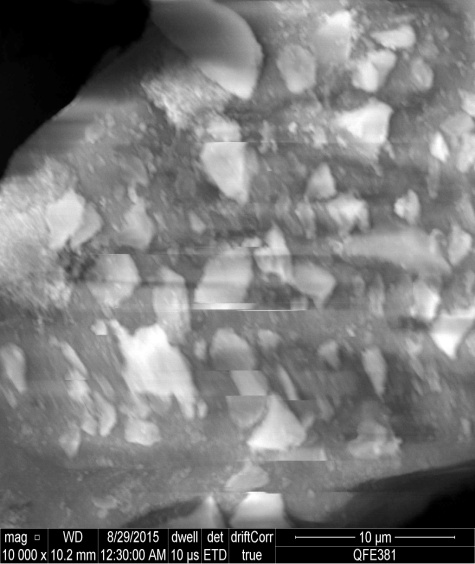

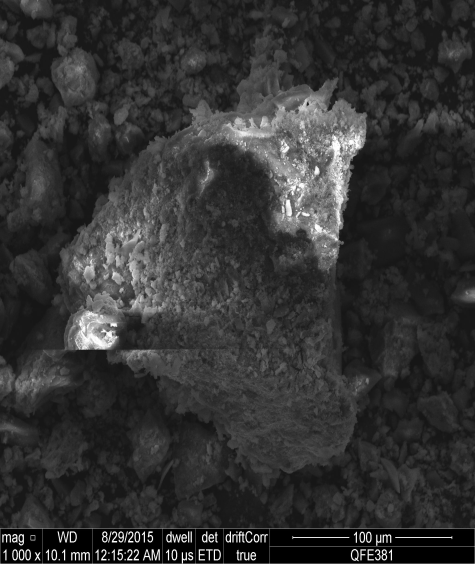

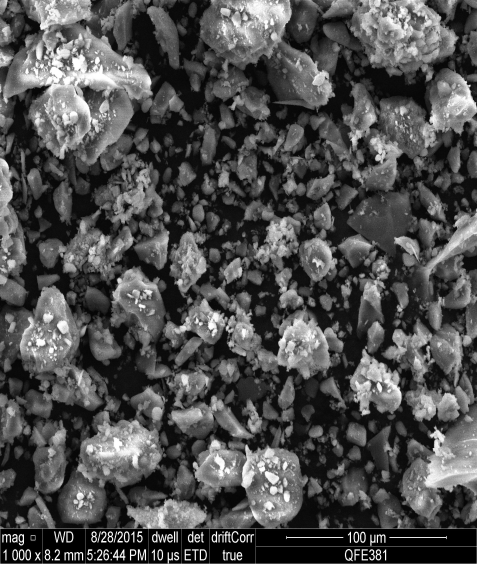

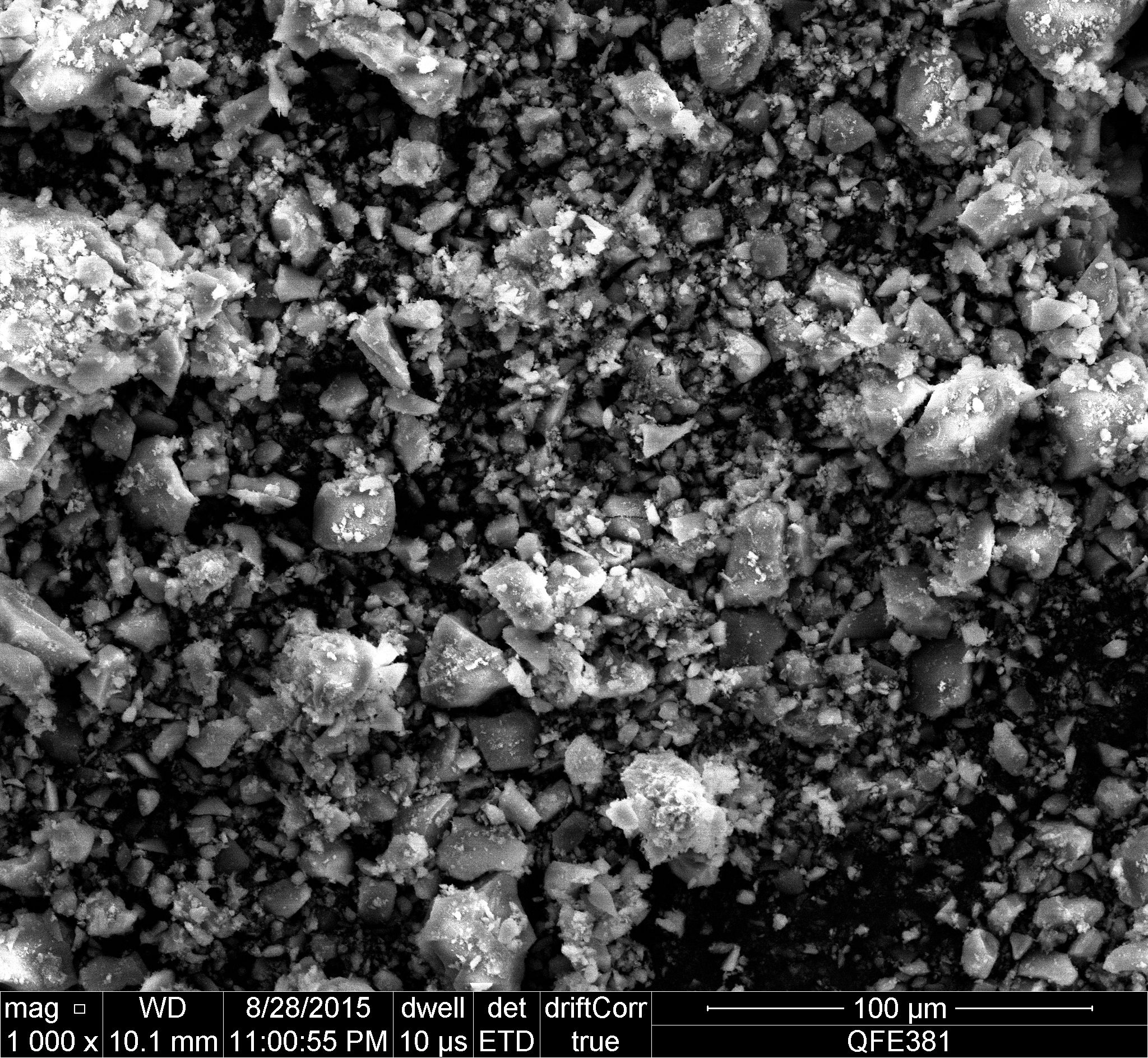

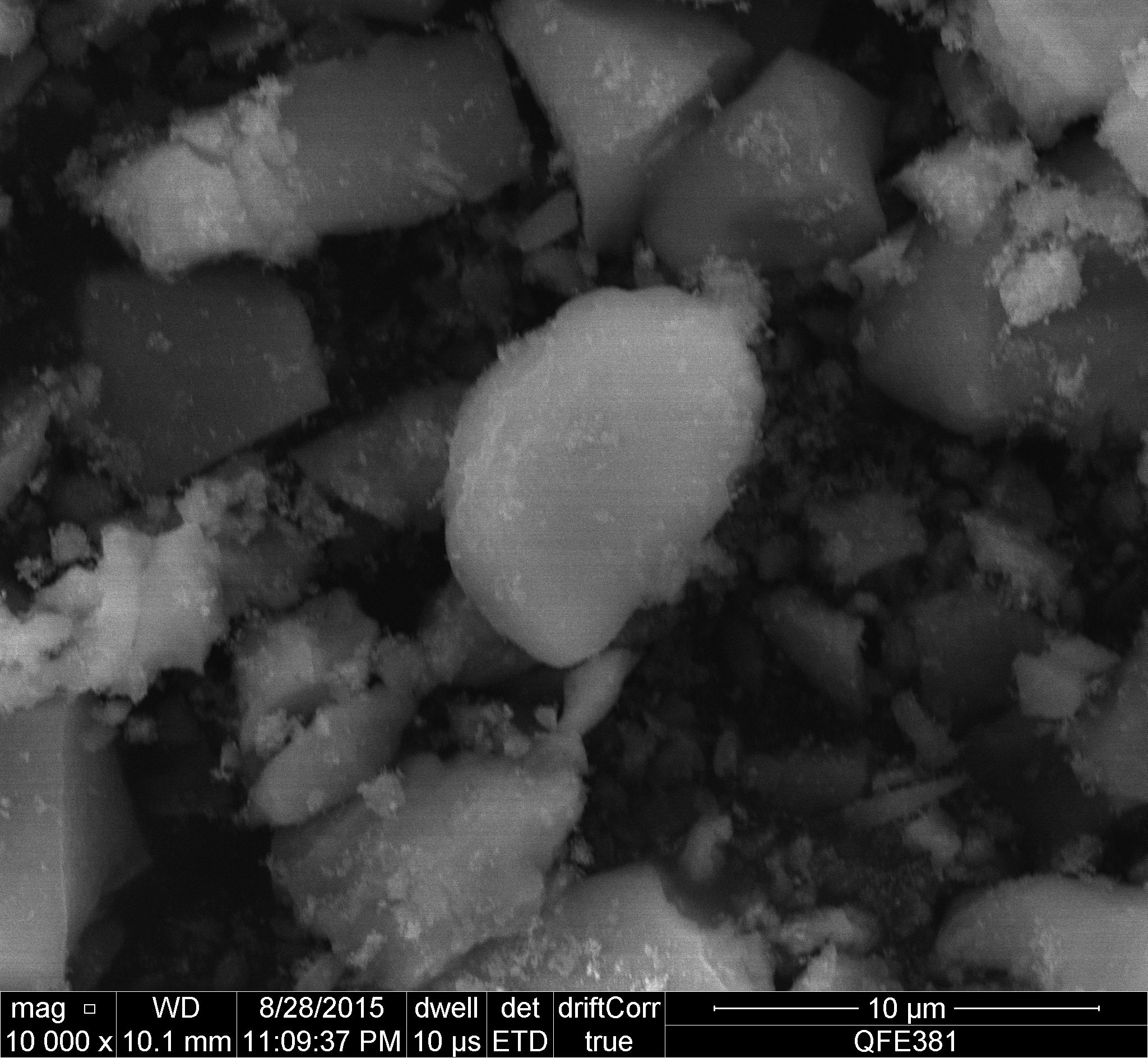

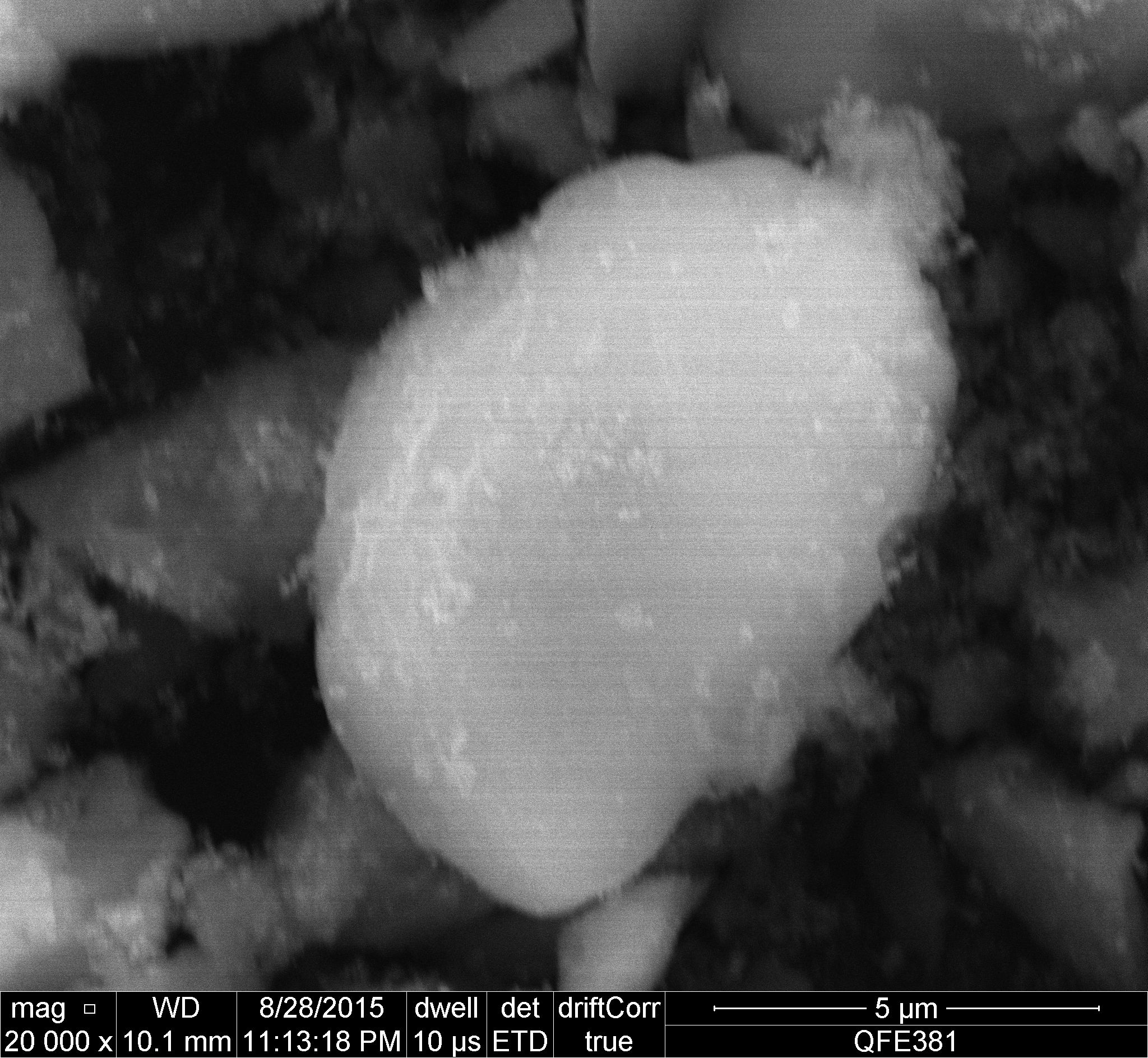

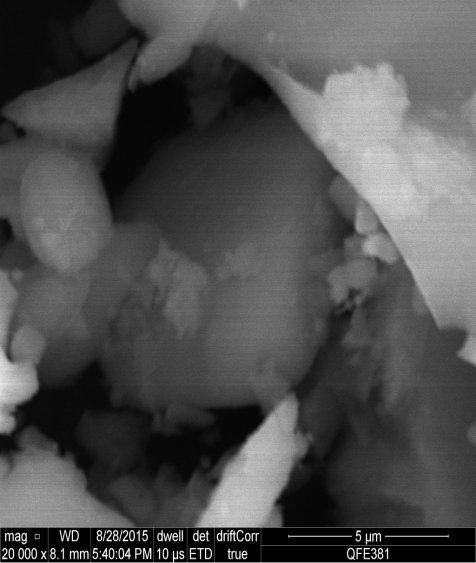

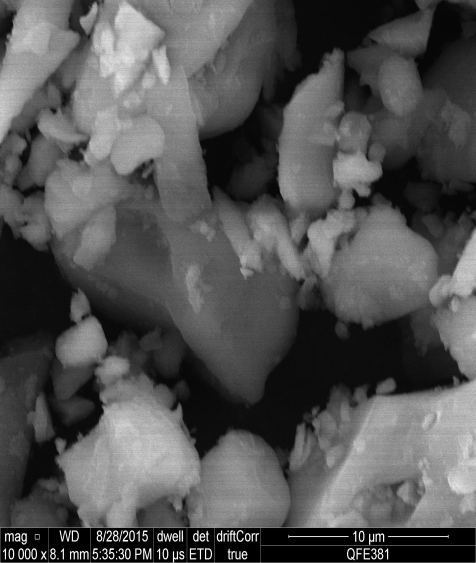


**(C)**

**(A)**

**(B)**

**Figure (S7):** SEM images of (A) 2% Cu/AC550, (B) 6% Cu/AC550 and (C) 15% Cu/AC550 with magnification 1000 (left), 10000 (middle) and 20000 (right), respectively.

**Figure (S8):** The effect of different particle sizes on the catalytic activity of 6% Cu/AC550, 250-425 µm pellets (solid line) and powdered form (dash line). Reaction conditions: T= 180-300 °C; catalyst weight= 200 mg; He flow rate = 80 ml/min; WHSV: 12.1 h^-1^.
